# A Study on the Effectiveness of IT Application Education for Older Adults by Interaction Method of Humanoid Robots

**DOI:** 10.3390/ijerph191710988

**Published:** 2022-09-02

**Authors:** Sungwook Jung, Sung Hee Ahn, Jiwoong Ha, Sangwoo Bahn

**Affiliations:** 1 Department of Industrial and Management Systems Engineering, Kyung Hee University, 1732, Deogyeong-daero, Giheung-gu, Yongin 17104, Korea; 2 Department of Industrial Engineering, Seoul National University, Seoul 08826, Korea

**Keywords:** robot, education, interaction type, elderly, human–robot interaction

## Abstract

Education using humanoid robots can have a positive impact in many fields, including in medical or physical training. This study investigated the effects of robot interactions with respect to facial expressions, gestures, voices and their combinations on the education of the elderly regarding information and communications technology (ICT) from functional and emotional perspectives. In this study, the robot’s interaction methods were divided into four categories: (1) voice, (2) voice and expression, (3) voice and gesture, and (4) voice and expression and gesture. An experiment involving an educational application with a humanoid robot was conducted with a total of 15 elderly people over the age of 60. The effect of the humanoid robot’s interaction method on education was identified by means of subjective survey evaluation and practice performance data analysis, including error rate, task success rate, and number of retrainings. Through the experiment, functional and emotional aspects of effects were measured. The results showed that performance and perceived effectiveness were not significantly affected by the type of robot interaction, but the degree to which the robot felt like it had emotions, the degree to which the robot felt like a human, and the degree to which the robot was friendly were significantly different according to the interaction type employed by the humanoid robot. The best effect was achieved when voice and gesture were used together during tutoring. Recognizing that ICT education using humanoid robots increases interest and participation in education, such robots are concluded to be a suitable method for performing ICT education. In addition, when designing robotic interactions, the use of the robot’s voice and gestures together is expected to lead to greater anthropomorphism, resulting in a stronger relationship with humanoid robots.

## 1. Introduction

Although new IT devices are being introduced into the market, as well as new methods of interacting with devices using information and communications technology (ICT), their benefits are not equally distributed among users. Older people are presented with a greater digital divide, which refers to any uneven distribution of access, use, or impact of ICT compared to younger generations. This is because the elderly normally have difficulty following technological changes or learning how to operate new devices [1,2]. According to the National Information Society Agency, the level of digital informatization is the lowest among the elderly. This digital information gap with regard to the elderly creates severe social problems, such as segregation between generations, which subsequently result in depression and alienation among the elderly, in addition to the simple inconvenience of using ICT [3].

There are two types of digital divide. The first digital divide is related to the availability of technical infrastructure, and the second digital divide is caused by a lack of skills regarding the use of digital services [4]. The second digital divide mainly results in a gap between the elderly and the younger generation [5]. Age, among demographic factors, is known to be the most influential factor, and causes an increase in anxiety with respect to technology, and negative attitudes towards ICT, as well as a decrease in cognitive and psychological abilities like attention, memory and technology acceptance [5]. These characteristics keep the elderly from adjusting to cutting-edge technologies and learning the skills required to use devices.

Problems related to the digital divide can be solved through education [6]. In fact, various educational programs related to ICT have been being provided to the elderly, but issues have arisen in terms of their effectiveness. Lectures conducted at elderly welfare centers and cultural centers are often limited to one-time events, and the limited times and places of education lead to accessibility problems for the elderly. Additionally, in the case of older people, the phenomena of low self esteem or hesitation present obstacles to learning how to use digital devices from the people around them. Lack of confidence and perhaps the fear of embarrassing oneself are frequently reported obstacles to the use of ICT and the Internet among older adults [7,8].

According to previous studies, humanoid robots, the overall appearance of which is based on the human body structure, are used in various fields, ranging from daily life to medical care for the elderly [9]. Humanoid robots have been used as trainers for the elderly [10,11,12]. Physical training using humanoid robots could be advantageous for the elderly in terms of accessibility and acceptability [13]. Treatment through playing with humanoid robots has been shown to be effective for improving cognitive function and daily life abilities of elderly people with dementia [14], and the interactions between the elderly and robots have been shown to be effective for enhancing psychological stability and social participation among the elderly [15,16].

In this way, education using humanoid robots can provide positive effects not only in the areas of medical care or physical training, but also in many other applications. Several studies have proved the impact of the use of humanoid robots on education in the case of young students [17]. For example, the gestures performed by the robot can increase the learning achievement by directing the learner’s attention [18]; therefore, interacting with humanoid robots can enhance interest in learning and motivate learning [19,20]. However, with increasing age, there is a tendency to avoid or hesitate before interacting with robots [21], and the acceptance of robots among the elderly will be different from that of the younger generation. When the elderly adopt new technologies, devices or digital applications, it is necessary to provide the right form of training or education [22]. For this reason, interaction with humanoid robots among the elderly needs to be designed in consideration of their specific characteristics in order to enhance satisfaction and performance. Therefore, it is necessary to determine the modes of humanoid robot interaction that are appropriate for engaging the elderly to participate in education. The present study examined the effect of robot interactions such as facial expression, gesture, voice, and their combination on education for the elderly. To this end, an experiment was conducted using a humanoid robot as an education agent while controlling for other effects. Since the artificial voice or tone of the robot’s utterance may influence the result, the educational content was delivered by an instructor through the robot.

## 2. Literature Review

### 2.1. Functional Aspect in Education

Researchers have tried to determine the effectiveness of robots in education and training from a functional perspective. In those studies, it was shown that physical robots and graphically expressed virtual agents can be effective educational agents. Previous studies have been conducted to understand the effect of the facial expressions and gestures used by virtual agents when they are the main training assistant [23,24]. Moreno et al. [25] concluded that using a virtual agent that performed gestures during education was able to increase the learners’ attention, compared to using visual cues such as arrows. In their research, which investigated the influence of the agent’s instructional gestures, conversational gestures, and facial expressions on learning to understand, Ryu and Yu [26] found that the agent’s facial expressions and gestures directly affected learning comprehension. Other studies have focused on several aspects of robots, including facial expression, voice, and gesture. They concluded that the facial expressions or body gestures of the robot can create synergy in education, since paralinguistic cues from humanoid robots are able to deliver messages [27], and the robots can enhance the learning experience through the appropriate manipulation of interactions [28]. Saerbeck et al. [19] found that the interactions that used the robot’s facial expressions and gestures were the more effective for motivating learning and forming a bond with the robot than interactions without facial expressions or gestures. In contrast, Brown et al. [20] experimented with a robot using four interaction methods: (1) without robot, (2) with the robot interacting with voice only, (3) with the robot interacting with gestures only, and (4) with the robot using both. It was found that there was no significant difference in performance resulting from the interaction methods used by the robot, but that using the robot could still help learners to achieve improved concentration in education.

The facial expressions and gestures of humanoid robots and virtual agents in education are, in some respects, not similar [29,30], and it is agreed that non-verbal interaction affects learners during education. However, the effects of the interaction methods are controversial, and are affected by the purpose or context of the use of robots. 

### 2.2. Emotional Aspects in Education

When using robots for educational purposes, a long-term relationship is important, and acceptance of the robot can be seen as a major factor in this. Attitudes toward robots influence the tendency to accept or reject robotic devices [31]. In particular, anthropomorphism among the properties of humanoid robots is an important factor in user acceptance and long-term relationship with the robots [32,33], because it leads users to have a greater willingness to accept robots and to alleviate stress during their interactions with them by increasing familiarity, promoting social interaction, and making users more involved [34].

Although its effectiveness was dependent on the degree of induced anthropomorphism in learning materials, and the effect varied according to the students’ prior knowledge level, it was found that anthropomorphic features in robots could improve performance during education for the students [35]. Baylor and Kim [36] also confirmed that the agent’s facial expression could promote the emotional bonding of learners with the robots. Indeed, studies related to interaction design have been performed with the aim of enhancing the anthropomorphism of educational agents, but there is still discordance in the conclusions regarding the effect of the interaction type on anthropomorphism. To enhance the anthropomorphic properties of robots, it is necessary to design human–robot interaction in an appropriate manner.

### 2.3. Research Questions

The voice of the robot can be an important factor influencing robot–human interactions in education [37]. Appropriate non-verbal interactions can also help students achieve improved concentration, increased motivation, and enhanced memory of vocabulary, but choosing an approach involving excessive interaction can also be counterproductive to education [38]. Numerous studies have provided evidence for the efficacy of non-verbal behaviors, including facial expressions, gaze, and gestures in improving learners’ comprehension [39,40,41]. However, there is a lack of research on ICT education for the elderly using robots.

In the present study, the following two questions were tested in a population of elderly participants with the aim of investigating the effect of different types of interaction.

Q1. Does the interaction type affect the performance and perceived effectiveness of ICT training among the elderly when the content controlled and the feedback of the robot is presented effectively? 

Q2. Does the interaction type affect emotional aspects such as anthropomorphism and satisfaction in ICT training among the elderly when the content is controlled and the feedback of the robot is presented effectively?

To determine the answers to these questions, an experiment was conducted involving elderly participants interacting with a humanoid robot as a main training assistant, but with the content provided by the voice of human instructors, assuming that future robots for ICT education will be fully automated to provide both content and feedback. The content was a controlled variable in this experiment. Four types of interactions were defined to compare the impacts on the education: (1) voice only, (2) voice and facial expression, (3) voice and gesture, and (4) voice, facial expression, and gesture.

## 3. Materials and Methods

### 3.1. Apparatus

**Figure 1:**
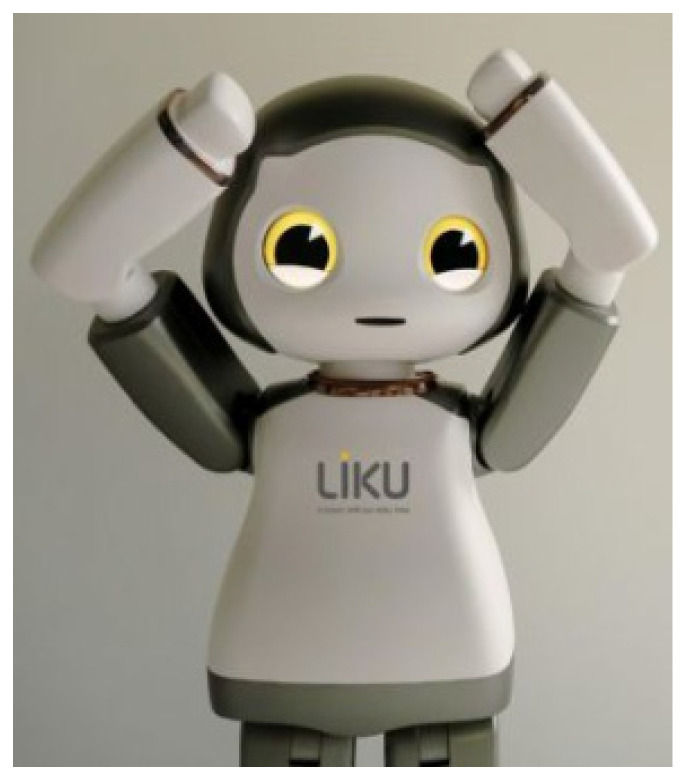
Experimental environment.

A humanoid robot, Liku^TM^ (TOROOC, Seoul, Korea), was used in the experiment (Figure 1). The robot, which looks like a child, can move its head, arms, and legs, and can perform a variety of facial expressions through a display located in the eyes. The height and weight of the robot are 60 cm and 2.5 kg, respectively.

The smartphone used in the experiment was a Samsung Galaxy S7 edge. In South Korea, most elderly people use mobile phones operating Android OS. The most widely represented Android phone in their generation was selected for the experiment for this reason. The size of the smartphone, the Galaxy S7 Edge, is 150.9 mm long and 72.6 mm wide.

### 3.2. Interaction Types of the Robot

The robot interacted through voice, facial expression, and gesture. In this experiment, there were four combinations of these interactions employed: (1) voice only, (2) voice and facial expression, (3) voice and gesture, and (4) voice, facial expression and gesture. The voice interactions were included in all experiments to clearly provide the actions and information to be performed by the subjects.

For the voice feedback, short and simple sentences were selected, because the cognitive load of the elderly increases when sentences spoken by robots are long or complex [42]. During the training session, the participant was praised in the case of success in performing the task and in the opposite case, received encouragement from the robot. Examples of the voice feedback are shown in Table 1.

### 3.3. Gestures and Facial Expressions of the Humanoid Robot

**Figure 2:**
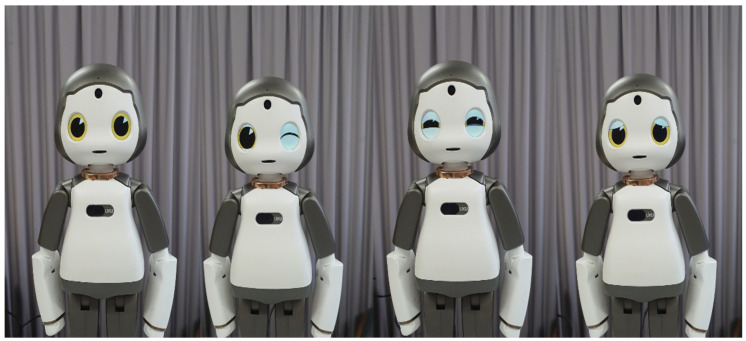
Facial expressions.

Table 2 shows the details of the robot’s gestures and facial expressions used during training and the experiment. Appropriate gestures and facial expressions were provided by the robot in response to the context. The facial expressions are illustrated in Figure 2: an expressionless face, and faces that are winking, smiling, and sad, are shown in order from left to right. The robot provided instructions and feedbacks for each interaction type. In the case of interactions that excluded facial expressions, consisting of voice only or voice and gesture, the robot maintained the default face.

### 3.4. Target Application

The target application was KakaoTalk Version 8.5 (Kakao Corp., Seoul, Korea), which is the most popular messaging app in Korea. Considering that lectures on the KakaoTalk for the elderly have been being conducted at welfare centers and community centers, KakaoTalk was regarded as being the most suitable application for the purposes of this experiment among the elderly.

Prior to this experiment, 15 elderly people were surveyed on the use of KakaoTalk, and four experts on teaching the use of smartphone apps and computers to the elderly were interviewed in order to select appropriate functions in the messaging application. On the basis of these interviews, six functions were selected, in consideration of the requirements, usefulness, frequency of potential use, and difficulty of the functions. Table 3 shows the six functions and the corresponding sub-functions.

**Figure 3:**
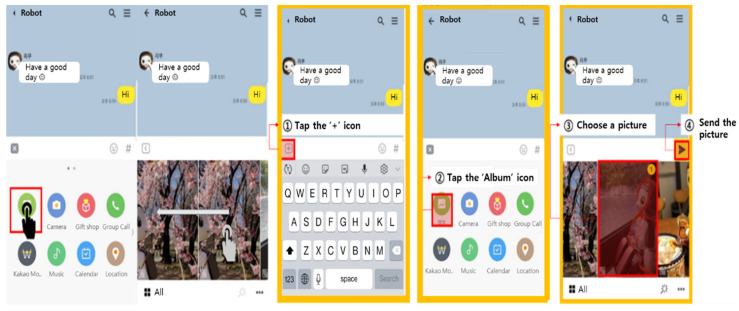
Training materials indicating how to find and share a picture.

During the training session, the materials were provided in the form of a visual aid, including the position of the buttons and the touch area, along with animations, in order to assist with understanding. An example of the materials is shown in Figure 3.

For an efficient experimental design, an orthogonal array was utilized to match each interaction type and the function of the application. The order of function execution was the same for every participant, because the difficulties of each function are different. However, the order of interaction types for each function was randomized in order to remove the learning and carryover effect. The participants performed each function as a task, and each task was paired with an interaction type.

### 3.5. Participants

An experiment was conducted with a total of 15 elderly people aged from 60 to 85 (male: 4, female: 11, average age: 66.8 years ± 6.7). All participants were familiar with using KakaoTalk, and the average number of years of use was 4.9 (±2.2 years). However, the participants’ usage was limited to basic functions such as receiving and sending messages. They also reported that they experienced difficulties using KakaoTalk, with the major difficulties they encountered including hard-to-understand terms, constant forgetfulness, and confusion with other phone features.

### 3.6. Experimental Environment

**Figure 4:**
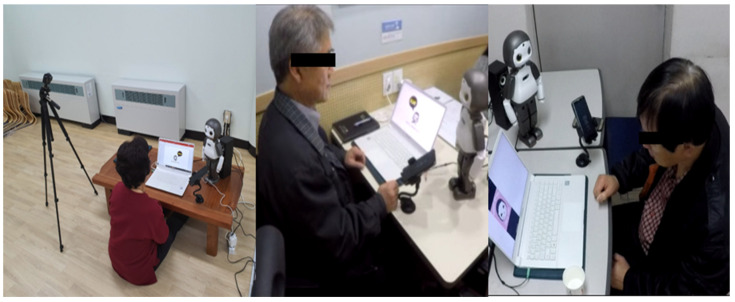
Experimental environment.

To minimize the effect of the experimenter, the experimenter conducted the experiment from a separate room (Figure 4). The participants were trained by the robot’s coaching and visual aids presented through a laptop. They performed the task using the given smartphone. The font size and keyboard type were set to be the same as the participant’s own smartphone, so as not to require any adjustment time and to minimize factors that could affect the performance. Feedback and retraining comments were provided remotely by the experimenter, and the gestures and facial expressions of the robot were also remotely controlled using an emulator application.

### 3.7. Experimental Procedure

**Figure 5:**
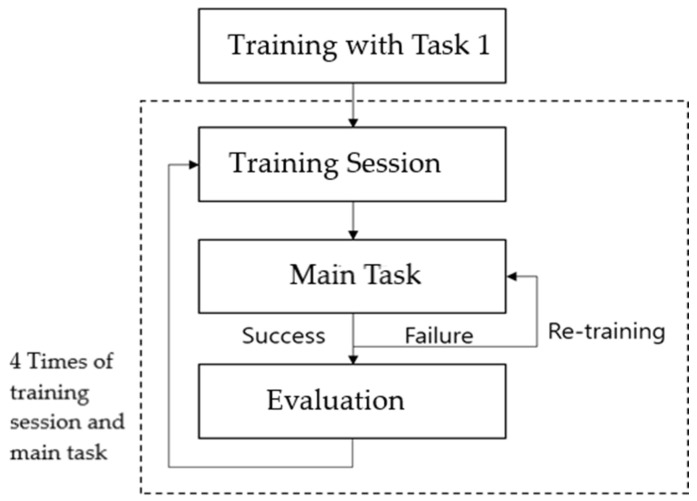
Experimental procedure.

All participants performed task 1 first, in order to adapt to the tutoring of the robot; the participants checked a friend’s profile and created a new chat room. After task 1, other tasks were performed during the training session, one at a time. The participant performed the corresponding task. In the post-educational practice situation, feedback was provided according to success and failure, and if the subjects failed the practice, retraining was conducted through feedback until they succeeded in the task by themselves. If the participant succeeded in the task, a questionnaire was provided, and this was repeated four times. The training time per task was around 4 min, and the interaction types matched in advance were randomly applied to the interactions for each training. The overall experimental procedure is shown in Figure 5.

### 3.8. Measures

All participants performed task 1 first to adapt to the tutoring of the robot; the participants checked a friend’s profile and entered a chat room. As mentioned in the research questions, two aspects of the effects were measured: functional and emotional aspects. For the functional aspect, performance and effectiveness were measured, while satisfaction, fun, and anthropomorphism were measured for the emotional aspect.

Questionnaires were developed to measure satisfaction and the perceived anthropomorphism of the robot as a function of the interaction types employed by the humanoid robot. First, a questionnaire used for the Unified Theory of Acceptance and Use of Technology (UTAUT), which aims to determine users’ intentions towards new information systems and their subsequent usage behavior, was adopted. Second, other questionnaires for measuring the degree of anthropomorphism were also referred to in order to develop the questionnaires used in this experiment.

Questions regarding anxiety, enjoyment, and attitude toward the use of robots in the UTAUT questionnaire were included in order to measure satisfaction in the questionnaire, while questions about learning motivation, trustworthiness, human-likeness, and learning interventions were included in order to measure anthropomorphism. In addition, items such as degree of enjoyment of learning and helpfulness with concentration, which were used in previous studies identifying the effect of robot interaction on learning [20], were additionally reviewed.

The questionnaires were selected in consideration of the fact that the purpose of this experiment was to understand the effect of tutoring according to the interaction types employed by the robot. The survey consisted of 10 questions, comprising seven questions related to satisfaction regarding educational effect and three questions related to the anthropomorphism of the robot. To verify the possibility of resolving the gap in digital information level among the elderly, three questions related to the digital information gap were added following the experiment. All questions were measured using a 7-point Likert scale.

In addition to the subjective questionnaires, task performance was measured quantitatively for research question 1: the numbers of touch errors, retrainings, and successes were collected through camera recording.

## 4. Results

To understand the effect of the tutoring provided by the robot according to the interaction type employed, the results of questionnaire items and quantitative measurements were analyzed. To investigate the previously stated research questions, the effects were analyzed from functional and emotional perspectives. The analysis was performed using the IBM SPSS Statistics 25 and R programs. All statistical tests were performed at the level of significance α = 0.05.

### 4.1. Descriptive Statistics

**Figure 6:**
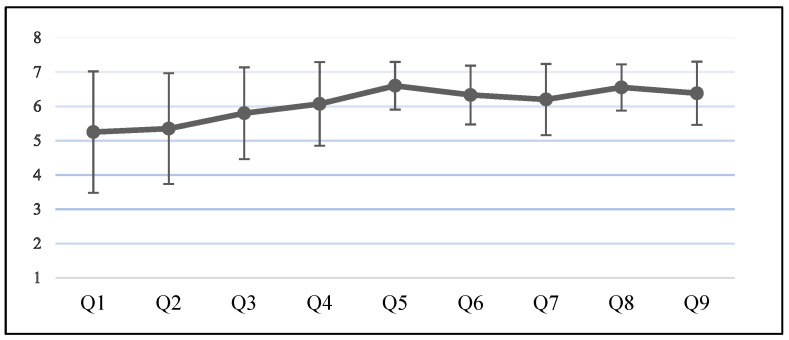
Descriptive statics of anthropomorphism and tutoring effect of the humanoid robot (N = 60).

A descriptive statistical analysis was performed to determine the effect exerted by the robot on tutoring. The participants gave the highest scores for their satisfaction regarding the ability of the robot to provide tutoring, which showed the least variance in responses (Table 4 and Figure 6). The question asking whether the robot had emotions received the lowest score with the largest variance. The participants provided high scores regarding satisfaction and perceived effectiveness, and relatively low scores regarding anthropomorphism.

### 4.2. Subjective Evaluation according to Interaction Type

The subjective evaluation addressed the degree of anthropomorphism, satisfaction, and the perceived effectiveness of the tutoring delivered by the humanoid robot. All scores for each question were non-normally distributed, so non-parametric analysis was applied in this section. The Kruskal–Wallis test was performed to understand the differences in subjective evaluation according to the interaction type. The results of the test indicated that there were no significant differences in perceived effectiveness. This means that the interaction type of the humanoid robot did not affect functionality. This result is consistent with the quantitative performance results described below.

To figure out the effects of the interaction type on the emotions of the participants, questions related to anthropomorphism and satisfaction were analyzed. The items related to anthropomorphism, including the degree to which the participant felt familiar with the robot, the degree to which the robot felt like a human, and the degree to which the robot seemed to possess emotions exhibited significant differences according to the interaction types (Table 5). Additionally, there was a slightly significant difference in satisfaction. The items for measuring satisfaction addressed the degree of interest and general satisfaction. Although the *p*-values were higher than the significance level α = 0.05, they were lower than 0.1. Hence, it was confirmed that there was to some extent a significant difference in the satisfaction.

**Figure 7:**
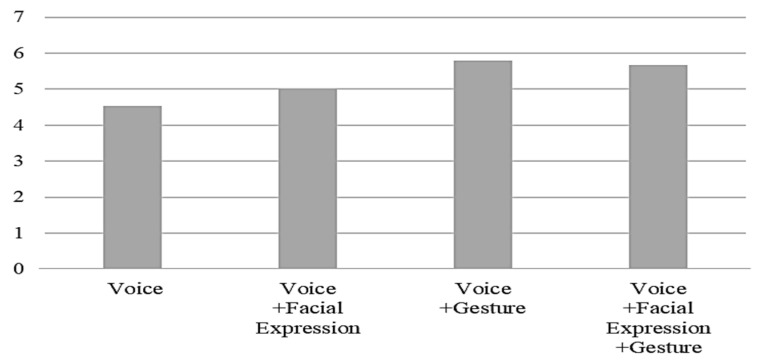
The degree to which the robot was perceived to have emotions by interaction type.

**Figure 8:**
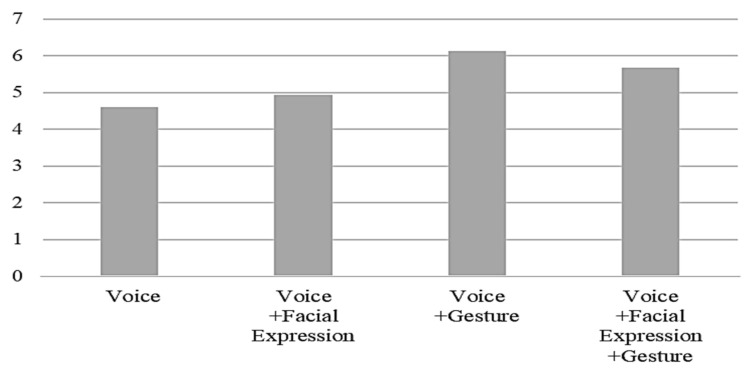
The degree to which the robot felt like a human by interaction type.

**Figure 9:**
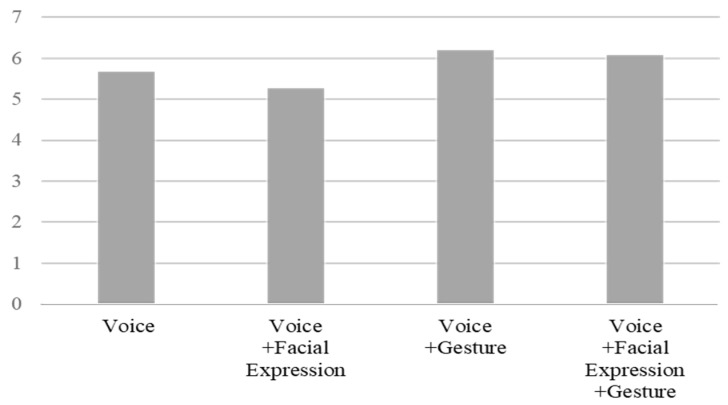
Degree to which the participants felt familiar with the robot by interaction type.

The questions related to the anthropomorphism of the humanoid robot, including the degree to which the robot was perceived to have emotions, the degree to which the participants felt familiar with the robot, and the degree to which the robot felt like a human, were scored highly in the interactions using robot gestures together with a voice compared to the voice only interaction (Figure 7, Figure 8 and Figure 9). It seems that gesture is the most influential factor in the degree of anthropomorphism accepted by the users.

### 4.3. Task Performance according to the Interaction Type

The subjective evaluation consisted of an assessment of the degree of anthropomorphism. Similar to the subjective evaluation, the measured task performance did not follow a normal distribution, so non-parametric tests were conducted to determine whether there was a difference in the number of touch errors and retrainings, as well as whether the training was successful, according to the interaction method. The analysis was performed based on the data from a total of 14 participants. The results showed that there were no significant differences in any of the quantitatively measured indicators related to performance. Detailed descriptions of each measurement are described in the following sections.

#### 4.3.1. Touch Errors according to the Interaction Type

A Kruskal–Wallis test was performed to determine the difference in the number of touch errors according to the interaction type. The results showed that there was no significant difference in the number of touch errors as a function of interaction method (Table 6).

#### 4.3.2. Number of Retrainings according to the Interaction Type

To determine the difference in the number of retraining according to the interaction method, the Kruskal–Wallis test was performed. The results of this analysis suggested that there was no significant difference in the number of retrainings according to the interaction method (Table 6).

#### 4.3.3. Success Rate according to the Interaction Type

Cross-tabulation analysis was performed to determine whether the interaction method affected the success of the practice. The success of the practice was the number of times that the practice was performed by participants themselves in a single training, and the analysis results show that the interaction method did not affect the success of performing the task (Table 7).

### 4.4. Results of a Survey on the Digital Divide

For the questions related to resolving the digital divide, the robot was evaluated positively, as shown in Table 8. The elderly people strongly agreed on the question as to whether robots could be helpful for providing tutoring in the use of other IT devices, and they also indicated that the training was not burdensome or uncomfortable. Additionally, the participants agreed that education provided by robots could help to form social relationships. Hence, it is expected that humanoid robots could be of great help in bridging the digital divide.

## 5. Discussion

The overall score in terms of functionality was high (higher than 6.0), so it can be inferred that using robots for the education of the elderly with respect to ICT can be profoundly useful. However, the quantitatively measured performance did not differ with the interaction type, and the answer to the first research question is that there is no significant effect of the interaction type on either perceived effectiveness or performance. The reasons for the insignificant differences are thought to be related to the content and the feedback. Throughout the whole experiment, the educational materials were controlled by the instructor, and feedback was provided at a suitable time. Hence, appropriate content and feedback are the key factors affecting performance when educating and training the elderly. Unlike in previous studies on young children, the interaction type did not affect the performance, which could be a result of the difference between young children and the elderly. This indicates that children and the elderly do not possess the same characteristics, and should therefore be treated differently. Furthermore, the tasks in this study did not require a high degree of visuospatial ability. Hence, robot gestures that might be helpful for performing certain types of task, such as pointing in a direction or mimicking human movement, were limited to the role of communicating emotional empathy with the participants in this study. 

The second research question was about whether the interaction type had an effect on the anthropomorphism from an emotional perspective, and the answer was yes. There were significant differences in anthropomorphism as a function of interaction type. Gesture played a key role in increasing the degrees of satisfaction, familiarity, and human likeness. According to neuroscience research, humans tend to perceive anthropomorphic properties in moving objects such as moving robot arms. Previous studies have reported that the gestures of the robot carried a greater degree of anthropomorphism, leading to positive intention [34,35,36,37,38,39,40,41,42,43,44]. Moving robots can evoke emotional responses to a greater degree than static robots. The robots were regarded as being more anthropomorphic when using co-linguistic gestures during the interaction. The participants tended to perceive the robot to be more familiar and realistic, and were more willing to use the robot with gestures in the future, compared to when the robot provided dictation without gestures, even when the robot provided content that was not aligned with the gestures, negatively influencing task performance.

Therefore, it can be inferred that it is more effective for robots to use natural gestures along with voice, in order to increase the degree of anthropomorphism of the robots in the tutoring of the elderly. With increasing age, the intention to use the robot decreased, so the emotional connection between the robot and the elderly is an important factor in participating in interaction with the robot [45]. This means that the gestures of the robot can effectively reduce the psychological distance between the elderly and the humanoid robot, improving participation not only in education, but also in interaction with the robot. Although it was not significantly different among the robot interaction types, the satisfaction with the education and the score of the intention to re-join the education were high for interaction including gestures. Therefore, the use of robots that perform gestures along with speaking may help the elderly become familiar with the robots used in humanoid robot education.

In human computer interaction, user engagement is one of the most important concepts, not only for the design and implementation of basic interfaces, but also to enable more sophisticated interfaces to which a user is better able to adapt [46]. When it comes to designing humanoid robots, user engagement is also important from the same point of view, because the users who interact with the robots should be willing to engage with the robots in order to achieve effective training and education in the long term. Therefore, when considering the results of this study, the use of appropriate gestures to increase the degree of anthropomorphism could be an effective way of encouraging user engagement with the robots. Anthropomorphism can be a critical factor affecting educational effectiveness from a long-term perspective, too. This result is also consistent with a previous study on anthropomorphism and engagement in voice assistants [47]. As found in previous studies, intimacy between users and robots can be helpful in education using robots.

It can be concluded that it is possible to bridge the digital information gap among the elderly. In the case of the elderly people who participated in this study, the degree to which they believed that robots were able to educate them, and the degree to which they thought they could learn from robots were both sufficiently high. Overall satisfaction with education and scores of intention to re-engage in education were high for all interaction types. This means that humanoid robots are able to effectively educate the elderly, even in the long run. As a result of survey evaluations and interviews related to bridging the digital information gap, most of the respondents said that the robot’s training was easy to understand and fun, and that it would be possible to receive sufficient education for other IT devices through the robot. In addition, a high percentage of participants said that the training they received from the robot was good, because they did not feel ashamed about failure during the practice, and they were able to receive retraining without burden. In questions related to social participation, a high percentage of respondents said that their confidence in the use of the application functions improved due to the education provided by the robot, and that they would be able to have more conversations with their acquaintances using the messaging application. The advantage of robots is that they can deliver education repeatedly, without the restriction of use. Therefore, educational humanoid robots can solve problems with respect to the low accessibility to existing informatization education, and alleviate the negative psychological factors for the learning of the elderly, thereby reducing the digital information gap experienced by the elderly.

In this study, the facial expressions and gestures of the robot were limited in range, because the experiment was conducted using a prototype robot. If facial expression were not limited to the movement of the eyes, and more diverse facial expressions were implemented in the robot, then facial expression may affect the effectiveness of education received from the robot. Therefore, in future studies, it is necessary to understand the effect of the methods of interaction used by the robot on the educational outcomes on the basis of a more diverse set of gestures and facial expressions. Finally, this study cannot be regarded as representing all of the various characteristics of the elderly, because the number of subjects was small. Nevertheless, although representativeness of the elderly may be lacking, it is meaningful as a basic study on the possibility of education using robots among the elderly. All participants were fairly familiar with the KakaoTalk application, and their cognitive and physical characteristics were not considered. Therefore, future research should classify elderly people into several groups according to the aforementioned criteria and conduct experiments on them. In addition, as the elderly’s proficiency in using IT applications is improving amid the acceleration of aging, it is thought that it would be meaningful to analyze this difference by dividing the age groups into those in their 60s–70s, and those who are older.

## 6. Conclusions

This study classified the interaction types of humanoid robots into (1) voice, (2) voice and facial expression, (3) voice and gesture, and (4) voice and facial expression and gesture in order to understand the differences in the educational effect for the training of smartphone use among the elderly with respect to these interaction types. On the basis of the results of this study, it can be concluded that the degree to which the robot feels emotional, the degree to which the robot feels like a human, and the degree to which the robot is friendly differed significantly depending on the interaction type employed by the humanoid robot, and the greatest effect was achieved when voice and gesture were used together during tutoring. As it was recognized that ICT education with the use of humanoid robots among the elderly increased interest and participation in education, this can be considered to be a suitable method for ICT education. Additionally, the degree of anthropomorphism, which results in a more powerful relationship between the learner and the humanoid robot, is expected to be high when designing robot interactions to employ voice and gestures together. 

Studies have been conducted with the aim of understanding the educational effects of robot interaction methods. However, existing research was focused on grasping the educational effect when robots were used as an auxiliary tool in educational environments, and a study in which the robot took over the role of the actual teacher and conducted both training and the post-training practice session had not previously been conducted. In addition, no research has been conducted with the aim of understanding the educational effect of the interaction methods employed by robots among the elderly. This study is meaningful because it determines the possibility of education for the elderly using humanoid robots and the educational effect of the interaction methods employed by the robot. The results of this study are expected to serve as a useful guideline for the development educational humanoid robots for the elderly.

## Figures and Tables

**Table 1:** Examples of voice feedback.

Success	Failure
Well done.	Was it a little difficult? It’s okay. I will tell you again
It is correct. Will you be able to do well alone next time?	Can’t you remember? It’s okay. I will tell you again.

**Table 2:** Examples of gestures and facial expressions of the humanoid robot.

	Details
General gestures	(1) Raise and lower both arms diagonally
	(2) Raise and lower left and right arm alternatively
	(3) Raise and lower with one arm bent
	(4) Raise and lower one arm
General facial expression	(1) Smile
	(2) Wink
	(3) Blink
	(4) Concentrating
	(5) Sad
Feedback gestures	(1) Nod and clench a fist (in cases where tasks were successful)
	(2) Raise hands above head, shaking the body (in cases where tasks were successful)
	(3) Shake head from side to side, placing both hands on its chest (in cases where participants failed the task)
Feedback facial expression	(1) Smile (in cases where tasks were successful)
	(2) Sad (in cases where participants failed the task)

**Table 3:** Selected functions and sub-functions of the KakaoTalk app.

Task	Function (Sub-Function)
1	Creating a chat room (search a specific person/check profile image/create a new chat room)
2	Sending and saving pictures
3	Forwarding messages or pictures
4	Additional features of chat rooms (turn off notifications and invite another person to an existing chat room)
5	Pinning a specific chat room on the top
6	Deleting sent messages

**Table 4:** Descriptive statistics of anthropomorphism and tutoring effect of the humanoid robot (N = 60).

Item		Mean	SD
Anthropo-morphism	Q1. Did you feel that the robot had emotions?	5.25	1.772
	Q2. Did the robot feel like a human?	5.35	1.614
	Q3. Did you feel familiar with the robot?	5.80	1.338
Satisfaction	Q4. Was the tutoring interesting?	6.07	1.219
	Q5. Were you generally satisfied with the tutoring?	6.60	0.694
Perceived effectiveness	Q6. Could you understand the content the robot provided well?	6.33	0.857
	Q7. Could you focus on the tutoring?	6.20	1.038
	Q8. Do you think that you can learn through the robot?	6.55	0.675
	Q9. Do you think robots can educate?	6.38	0.922

**Table 5:** The results of the Kruskal–Wallis test on the subjective evaluation according to interaction type.

Item		Kruskal–Wallis H	*p*-Value
Anthropo-morphism	Did you feel that the robot had emotions?	8.921	0.030 *
	Did the robot feel like a human?	11.38	0.010 *
	Did you feel familiar with the robot?	8.368	0.039 *
Satisfaction	Was the tutoring interesting?	7.620	0.055
	Were you generally satisfied with the tutoring?	6.511	0.089
Perceived effectiveness	Could you understand the content the robot provided well?	0.902	0.825
	Could you focus on the tutoring?	1.833	0.608
	Do you think that you can learn through the robot?	2.129	0.546
	Do you think robots can educate?	3.886	0.274

* *p*-value ≤ 0.05.

**Table 6:** Kruskal–Wallis test on the number of touch errors and retrainings by interaction type.

Measurement	Interaction Method	N	Mean Rank	df	$\chi^{2}$	*p*-Value
Touch errors	Voice	14	28.00	3	0.328	0.955
	Voice + Facial expression	14	30.54			
	Voice + Gesture	14	27.64			
	Voice + Facial expression + Gesture	14	27.82			
Retraining	Voice	14	27.07	3	0.709	0.871
	Voice + Facial expression	14	30.86			
	Voice + Gesture	14	28.79			
	Voice + Facial expression + Gesture	14	27.29			

**Table 7:** Results of the cross-tabulation analysis on success rate according to interaction type.

Interaction Type	Task Performance		$\chi^{2}$	*p*-Value
	Success	Failure		
Voice	10(71.4%)	4(28.6%)	0.876	0.831
Voice + Facial expression	8(57.1%)	6(42.9%)		
Voice + Gesture	9(64.3%)	5(35.7%)		
Voice + Facial expression + Gesture	10(71.4%)	4(28.6%)		

**Table 8:** Descriptive statistics of the items on bridging the digital divide.

Item	N	Mean	SD
Do you think training by robot could be helpful in the case of other IT devices?	14	6.3	2.11
Was the training by robot burdensome or inconvenient? (1 = very burdensome, 7 = not very burdensome)	14	5.7	2.37
Do you think that training by robot can help to build social relationships?	14	6.3	1.27

## Data Availability

The data that support the findings of this study are available from the corresponding author, upon reasonable request.
